# Valence without meaning: Investigating form and semantic components in pseudowords valence

**DOI:** 10.3758/s13423-024-02487-3

**Published:** 2024-04-02

**Authors:** Daniele Gatti, Laura Raveling, Aliona Petrenco, Fritz Günther

**Affiliations:** 1 Department of Brain and Behavioral Sciences, University of Pavia, Piazza Botta 6, 27100 Pavia, Italy; 2 Institut für Psychologie, Humboldt-Universität zu Berlin, Berlin, Germany

**Keywords:** Valence, Semantic memory, Distributional semantics, Pseudowords

## Abstract

Valence is a dominant semantic dimension, and it is fundamentally linked to basic approach-avoidance behavior within a broad range of contexts. Previous studies have shown that it is possible to approximate the valence of existing words based on several surface-level and semantic components of the stimuli. Parallelly, recent studies have shown that even completely novel and (apparently) meaningless stimuli, like pseudowords, can be informative of meaning based on the information that they carry at the subword level. Here, we aimed to further extend this evidence by investigating whether humans can reliably assign valence to pseudowords and, additionally, to identify the factors explaining such valence judgments. In Experiment 1, we trained several models to predict valence judgments for existing words from their combined form and meaning information. Then, in Experiment 2 and Experiment 3, we extended the results by predicting participants’ valence judgments for pseudowords, using a set of models indexing different (possible) sources of valence and selected the best performing model in a completely data-driven procedure. Results showed that the model including basic surface-level (i.e., letters composing the pseudoword) and orthographic neighbors information performed best, thus tracing back pseudoword valence to these components. These findings support perspectives on the nonarbitrariness of language and provide insights regarding how humans process the valence of novel stimuli.

**Supplementary Information:**

The online version contains supplementary material available at 10.3758/s13423-024-02487-3.

Valence—the pleasantness of a stimulus and whether it evokes positive or negative emotions (Warriner et al., 2013)—is one of the most prominent semantic dimensions. Its importance in our lives is evident, with it being fundamentally linked to the basic approach–avoidance behavior (approach pleasant stimuli, avoid unpleasant stimuli; Krieglmeyer et al., 2013). Also in the language domain, the seminal factorial analysis by Osgood et al. (1957) identified valence as the most important component of word meaning.

Being a central component of word meaning, it appears only reasonable that a word should be meaningful in order to have a (positive or negative) valence. Conversely, any given meaningless word stimulus should be classified as neutral. However, intuitively this does not always hold: would you rather buy a food product called “simmy” or “grawp”? Traditionally, psycholinguistic studies have investigated words as meaningful stimuli, with pseudowords (out-of-vocabulary strings of letters that are consistent with the orthographical rules of a given language) serving as supposedly meaningless fillers. Yet, from the perspective of a given speaker encountering a word for the first time, there is no practical and functional difference between a pseudoword, on the one hand, and a novel word or a nonencountered existing word on the other hand. Studying pseudowords can therefore provide valuable insights about how humans process novel stimuli. And indeed, recent studies provide evidence for semantic effects in pseudoword processing, with slower response times for pseudowords with denser semantic neighborhoods (Hendrix & Sun, 2021) and for pseudowords semantically more related to a prime word (Gatti et al., 2023). Additionally, to some degree, when explicitly asked to do so, participants can even generate pseudowords that convey a given meaning (Pugacheva & Günther, 2024). These findings clearly call the assumption that pseudowords are meaningless into question.

Since pseudowords are constructed from the same subword units as existing words (e.g., the letters composing the stimulus, the bigrams), one can exploit this information to investigate whether the surface-level form characteristics of pseudowords are indicative of valence (e.g., whether pseudowords including a given letter are perceived as more pleasant). Another interesting source of information is, in this case, semantics. Indeed, previous studies on existing words have shown that their valence can be predicted both from their surface-level form characteristics (i.e., whether they contain specific letters or phonemes; Adelman et al., 2018; Aryani et al., 2018) and from their subsymbolic meaning components in the form of their distributional vector dimensions (Hollis et al., 2017).

Distributional semantic models (DSMs) represent word meanings as high-dimensional numerical vectors induced from the words’ co-occurrence patterns in large amounts of natural language data (distributional vectors), with words that are used in similar contexts in natural language ending up with similar distributional vectors (i.e., as having similar meanings; for a review, see Günther et al., 2019). This approach is rooted in the classical distributional hypothesis, stating that the contexts in which a word appears are indicative of its meaning (Harris, 1954, but see also Wittgenstein, 1953). However, while classical DSMs are highly performing across a wide range of psychological tasks (e.g., Gatti et al., 2022; Günther et al., 2016; Marelli & Amenta, 2018), they are restricted to their training corpus and the words in it—a word needs to be present in order to have a distribution over contexts. To overcome this, Bojanowski and colleagues (Bojanowski et al., 2017) developed *fastText,* a DSM that can estimate the meaning of any character string by quantifying the distributional pattern of the subword information contained in it. Each string of letters is modeled as a sum of vectors representing its embedded *n*-grams (see Fig. 1). Thus, *fastText* can approximate the meaning of pseudowords (Gatti, et al., 2023; Hendrix & Sun, 2021), defined as the semantic patterns that an out-of-vocabulary letter string can elicit. This allows us to empirically test whether the semantic information captured by distributional vectors for pseudowords can be informative of their valence too, as is the case for existing words (Hollis et al., 2017).

**Fig. 1:**
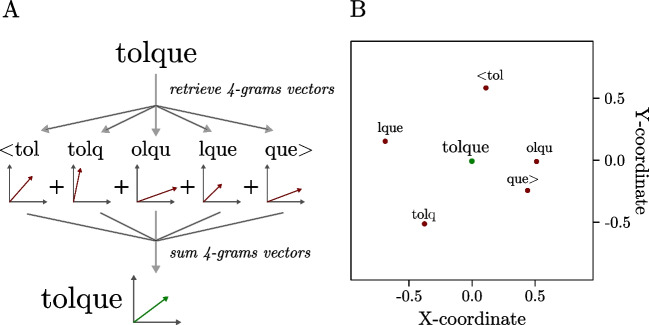
Graphical representation of how the *fastText* model used computes pseudowords vectors by retrieving subword 4-grams vectors and summing them up

Within this context, the present study aims to investigate whether humans can reliably assign valence to pseudowords and, additionally, to identify the factors explaining these judgments. In Experiment 1, we first trained a series of models on the Warriner and colleagues (Warriner et al., 2013) database to predict valence judgments for existing words from their form and meaning information (letters, bigrams, dimensions as emerging from a DSM, and their combinations). In Experiment 2 and Experiment 3, we applied these models to pseudowords (with no further or new training on these stimuli) and obtained several predicted valence indexes based on the relations between predictors and valence learned in Experiment 1. The observed valence of pseudowords was then compared to these predicted valence indexes. More briefly: we trained models on existing words and then applied these trained models to predict valence for entirely novel strings.

## Experiment 1

### Methods

#### Stimuli and procedure

From the valence norms provided by Warriner and colleagues (Warriner et al., 2013) we retrieved the 13,915 English words included and their valence. For the creation of this database, participants were shown one word at a time and asked to rate its valence on a 9-point scale (in the public database, the higher the value the more positive the valence). We removed words containing punctuation marks, capital letters or white spaces. The final set of stimuli consisted of 13,787 words.

Then, for each word, we counted the number of times each letter appeared in it. That is, for each word, we created 26 new columns (i.e., one for each letter of the Latin alphabet) and each cell was filled with a number indicating how many times within each word a certain letter appeared. The same procedure was applied to bigrams (i.e., possible combinations of pairs of adjacent letters including the start and the end of a word such as *<b* in *bus*; for a total of 676 possible bigrams) and to the starting and the ending letters.

Finally, for each word, 300 new columns were included. For each word, these cells were filled with the corresponding value of each of the 300 semantic dimensions retrievable from a distributional semantic model (DSM; see below).

#### Distributional semantic model

The DSM used here was *fastText* (Bojanowski et al., 2017; Mikolov et al., 2017) in its pretrained version available at https://fasttext.cc/docs/en/english-vectors.html (in the 2-million-word vectors version, trained with subword information). While the traditional distributional models can generate high-quality distributed semantic representations only for the words that are sufficiently frequent in the input data, *fastText* takes into account the subword information by computing word vectors as a sum of the semantic vectors for the 4-grams included in each word (the idea originally proposed by Schütze, 1993; and realized by Bojanowski et al., 2017). Crucially, this means that the word vectors can also be created for pseudowords, based on the subword units (i.e., n-grams) that they contain (see Fig. 1; Gatti et al., 2023; Hendrix & Sun, 2021).

The model was trained on the Common Crawl corpus using the Continuous Bag of Words (CBOW) method, an approach originally proposed by Mikolov and colleagues (Mikolov et al., 2013), with position-weights across 300 dimensions, character *n*-grams of length 4, and a window of size 15. When using CBOW, the obtained vector dimensions capture the extent to which a target word is reliably predicted by the contexts in which it appears. Word vectors were retrieved using the *fastTextR* R package (Schwendinger & Hvitfeldt, 2022).

#### Data analysis

Word valence in the Warriner and colleagues (Warriner et al., 2013) norms was predicted across three levels of increasing complexity: firstly, we estimated a linear model including only single-letter information, then, a model including bigram information (including also the starting and the ending letters that can be denoted as bigrams such as <a and a>), and finally, a model including semantic information in the form of *fastText* dimensions. Specifically, the linear model estimated for letters included 26 predictors:

$$Valence\sim a+b+\dots+z$$

For letters plus bigrams, the model included 754 predictors (26 letters + 676 bigrams + 26 starting letters + 26 ending letters):

$$Valence\sim a+b+\dots+z+aa+ab+\dots+zz$$

The full model included 1,054 predictors (the previous 754 predictors + 300 dimensions):

$$Valence\sim a+b+\dots+z+aa+ab+\dots+zz+Dim1+Dim2+\dots Dim300$$

### Results

For the letters model, we observed an *R*^2^ = .01 (*r* = .11) and an AIC = 45,677; for the letters plus bigrams we observed an *R*^2^ = .11 (*r* = .33) and an AIC = 45,292; and for the full model we observed an *R*^2^ = .65 (*r* = .80) and an AIC = 33,034.

As a subsequent sanity check, we also tested four other models differentiated by the sources of information included: one model included bigrams only, another dimensions only, and the other two included bigrams plus dimensions and letters plus dimensions respectively (i.e., all the possible combinations). For the bigrams only model, we observed an *R*^2^ = .11 (*r* = .33) and an AIC = 45,292; for the dimensions only model, we observed an *R*^2^ = .62 (*r* = .79) and an AIC = 32,949; for the bigrams plus dimensions we observed an *R*^2^ = .65 (*r* = .80) and an AIC = 33,034; and for the letters plus dimensions we observed an *R*^2^ = .63 (*r* = .79) and an AIC = 32,902. In this first experiment, we used *R*^2^ (instead of AIC) to evaluate the different models. Although the letters plus dimensions model had the lowest AIC (and thus appears as the best one in terms of explanatory power when considering model complexity), we nevertheless employed the full predictive power of the full model to select stimuli in the subsequent experiments. That is, in doing this we preferred to rely on the set of predictors that explained more variance. This decision was also made considering that the two models predicted valences with *r* = .98.

## Experiment 2A

In this second experiment, we use the model obtained in Experiment 1 to predict the valence of pseudowords and tested to which extent this converged with participants’ judgments on pseudoword valence. We collect these judgments through best–worst ratings. Contrary to classical Likert-scale ratings tasks, participants in this paradigm are shown *n* stimuli and are asked to indicate which one of them scores highest and lowest on a given dimension. Across all trials with different stimulus combinations, these best–worst judgments can then be converted into a continuous rating score (as for chess players’ Elo score).

### Methods

#### Participants

One hundred ninety-four U.S. participants (71 males, 116 females, seven nonbinary, *M* age = 39.1 years, *SD* = 14.5, age range: 18–80) were enrolled in the experiment through the Prolific online testing platform and were tested online. Twenty-seven participants were removed because they did not reach a good accuracy threshold when answering to the catch trials included (i.e., incorrectly classifying as positive or negative a nonpositive or nonnegative real word more than once in the whole task; see below). The final sample included 167 participants.

The sample size was determined a priori based on Hollis (2018, 2020). Specifically, in the best–worst scale rating technique employed here, the sample size depends on the overall number of items and the number of items presented to each participant. Previous studies demonstrate that presenting each item 30 times gives near-asymptotic performance in this kind of tasks (Hollis, 2018) and that presenting six items in each trial is optimal (Hollis, 2020).

In order to keep participants at a good compliance level we opted to keep the task relatively short, presenting 45 trials to each participant. Given a total of 7,500 trials (1,500 pseudowords presented for a total of 30 times, with six pseudowords in each trial), and the choice to have 45 trials per participants, the final sample size required was 167 participants.

All participants were native English speakers and were naïve to the purpose of the study. Informed consent was obtained from all participants before the experiment. The protocol was approved by the psychological ethical committee of the Humboldt University of Berlin (2020-47) and participants were treated in accordance with the Declaration of Helsinki. Participants were paid £1.80 for their participation in the experiment.

#### Stimuli

Pseudowords were created starting from the 28,730 words included in the British Lexicon Project (BLP; Keuleers et al., 2012). Words that contained punctuation marks, capital letters, or spaces were removed. The final set consisted of 28,475 words.

Using Wuggy (Keuleers, & Brysbaert, 2010), we automatically generated 786,013 pseudoword candidates. Starting from a given word, Wuggy allows for the generation of written polysyllabic pseudowords that obey a given language’s phonotactic constraints and that match its template in subsyllabic structure. That is, Wuggy generates highly word-like pseudowords but also stimuli that are not easily identifiable as related to existing words. Wuggy was set using its standard parameters—that is, orthographic English module, restricted match length of subsyllabic segments, restricted match letter length, restricted match transition frequencies, and match segments 2 out of 3.

Using the best-performing model from Experiment 1, we then predicted a valence value for each generated pseudoword. That is, for each pseudoword the frequency of each letter, the starting/ending letters, and the bigram was counted and values corresponding to the 300 *fastText* dimensions were computed. The computation of pseudowords’ semantic dimensions was made available by *fastText*’s ability to compute semantic representations by taking into account subword information by inducing semantic representations as the sum of the vectors of the letter *n*-grams associated with each word. That is, *fastText* computes the semantic representation of each string of letters as the sum of the vector of the full string (which should not exist for pseudowords) plus all the vectors of the 4-grams that compose it.

Because *fastText* is based on very large natural language corpora and might include some nonexistent character strings by mistake (e.g., as the result of typos), we systematically checked whether a “whole-pseudoword” vector was available in the generated pseudowords. In such cases, indeed, *fastText* could learn distributional patterns about these pseudowords as if they were meaningful elements, even if their occurrence was based on errors or typos. To overcome possible biases induced by the availability of this further piece of information, we removed such pseudowords, together with duplicates. The resulting trimmed set of pseudowords included 483,553 stimuli.

After collecting the 1,054 predictor values included in the full model (i.e., 26 letters, 26 starting letters, 26 ending letters, 676 bigrams, 300 dimensions) estimated in Experiment 1, using the *predict* R function we estimated a predicted valence index for each pseudoword from the abovementioned model estimates. The pseudoword with the lowest predicted valence was *xexen* with a predicted valence of 2.43 and the pseudoword with the highest predicted valence was *cupgel*^1^ with a predicted valence of 9.19. The mean predicted valence was 5.89, *SD* = .56.

**Fig. 2:**
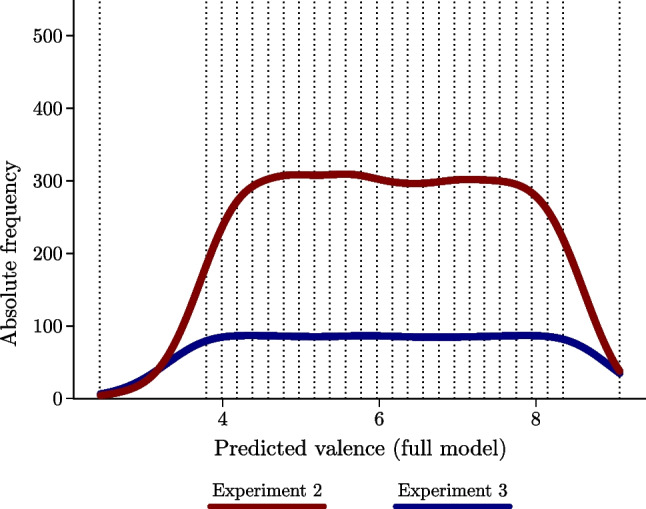
Distribution of the predicted valence of the 1,500 pseudowords included in Experiment 2 (red line) and Experiment 3 (blue line). Dotted lines indicate starting and ending points of each slice that was used to sample from the original set of pseudowords. (Color figure online)

Finally, 1,500 pseudowords were sampled so that the distribution of their predicted valence was as uniform as possible across the whole range of possible values. Specifically, firstly the pseudowords were ordered in descending predicted valence and the overall distribution was divided across 25 slices. The selection of the 1,500 final pseudowords was performed sampling randomly from the original sample of pseudowords and removing, across several rounds, pseudowords that were not readable in English or that were pseudocompounds (i.e., pseudowords composed of two existing English words, such as *cupgel*) until a flat distribution of 1,500 stimuli was reached. Stimuli selection was performed by A.P., L.R., and D.G. The first and the last slice corresponded to the two tails, with the left tail including pseudowords with a predicted valence ranging from 2.43 and 3.79, and the right tail including pseudowords with a predicted valence ranging from 8.35 and 9.07. The remaining 23 slices included a number between 50 and 61 pseudowords, with each slice covering around .2 points of the predicted valence range (see Fig. 2).

As described above, from these 1,500 pseudowords we built 7,500 trials composed of six pseudowords each, in order to have each pseudoword to appear 30 times within the overall set of trials. The items were assigned to the trials with a Latin square method, using the software provided by Hollis (2018).

Finally, we also built four catch trials. Specifically, we selected 24 existing words from the Warriner and colleagues (Warriner et al., 2013), of which four were highly positive (i.e., vacation, happiness, sunny, relaxation), four were highly negative (i.e., homicide, terrorism, virus, disaster), and the remaining 16 were neutral (e.g., column, episode, multiple, vertical). From these 24 words, we then built four trials, each comprising a positive word, four neutral words, and a negative word (e.g., sunny – semantics – multiple – telepathy – seal – virus). These trials were shown to all the participants, and since the most positive and the most negative word could be easily identified, they were used to remove the participants from the analysis for low compliance with the task.

#### Procedure

At the beginning of the experiment, participants were instructed that they would be shown several (new) words that could differ in the emotional response they evoked: Some could cause positive emotions, while others could induce negative ones. Participants were instructed to indicate which of the (new) words shown caused the most positive emotions (positive valence), and which caused the most negative ones (negative valence). They were also informed that many of the words they would be shown would not be part of the English vocabulary, but that they still might intuitively evokepositive or negative emotions. With this set of out-of-vocabulary items, the task was thus to focus on that potential emotional response and to evaluate it compared with the one induced by the other items presented. Following Warriner and colleagues’ (Warriner et al., 2013) instructions, participants were told to work at a rapid pace and not to spend too much time thinking about each item. Rather, they were asked to base their ratings on their first and immediate reaction as they read each item.

In each trial, participants were then shown six strings of letters presented in random vertical order (see Table 1 for an example of trial) in the center of the screen and were asked to judge which one had the most positive valence (as defined above) and which one had the most negative one (best–worst ratings; Hollis, 2018; Hollis & Westbury, 2018). Each participant was shown a unique set of 45 experimental trials (except for the additional four catch trials which were the same across all the participants). All the 49 trials were presented in random order. On average, the task took around 11 minutes to complete.

**Table 1:** Sample trial from Experiment 2A

Negative	Pseudoword	Positive
	tolque	
	divords	
	lurb	
	floal	
	ribnier	
	bureer	

Participants were asked to indicate which of the pseudowords shown elicited more negative and which more positive emotions. In this case we included two pseudowords that were generally rated as negative (i.e., divords) and positive (i.e., floal) and four neutral ones

#### Data analysis

The discrete best–worst judgments were transformed into continuous ratings scores (henceforth *valence indexes*) using the Value learning algorithm, the most robust scaling method among other possible candidates (Hollis, 2018). Generally, items will get higher scores if they are often picked as the most positive word, and lower scores if they are more often picked as the most negative word.However, even when a word is not picked in a given trial, this still provides the information that it is more negative than the most positive word, and more positive than the most negative one (for more details, see Hollis, 2018; Hollis & Westbury, 2018).

Firstly, we tested for split-half reliability. Participants were randomly divided into two groups, and the two observed valence indexes (one in each subsample) were obtained for each of the 1,500 pseudowords by applying the abovementioned procedure.

Then, we predicted these valence indexes in a linear model, using as the only fixed-effects predictor its predicted valence according to the full model tested in Experiment 1,^2^ (which, as described, was used to select the stimuli). This allows to investigate to what extent the valence effects predicted for words do generalize to pseudowords.

Additionally, in a follow-up analysis, we estimated six additional linear models, comparing the observed valence to the predicted valence from the other six models presented in Experiment 1 (namely, letters only, bigrams only, dimensions only, letters plus bigrams, letters plus dimensions, and bigrams plus dimensions). This second step allows to investigate whether other components (or combination of components) that are at play in predicting word valence can extend to pseudoword valence. Briefly, the analysis using the best model resulting from Experiment 1 allows to test whether humans do exploit the same (surface-level and semantic) processes that they use for words when they are asked to guess the valence of pseudowords; while the follow-up analysis allows to test whether other components could play a major role in the task at hand.

### Results

In a split-half reliability analysis, the two observed valence indexes were moderately correlated, *r* = .59, *p* < .001, demonstrating a moderate agreement between participants when it comes to judging the valence of pseudowords. While this index is somewhat lower than for standard valence ratings, it should be evaluated considering the complexity of the task at hand (see Table 1 for an illustration).

**Fig. 3:**
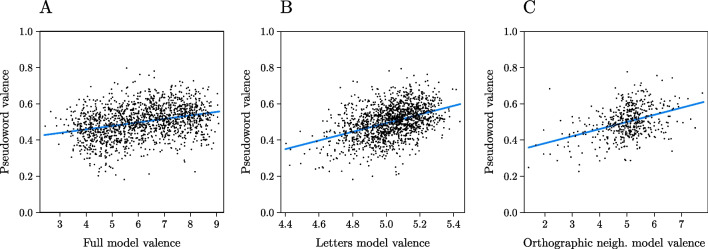
Plots showing: the relationship between pseudowords valence and the best performing model (i.e., the one comprising letters, bigrams, and dimensions) trained on words information (**A**), the relationship between pseudowords valence and the valence as predicted from the letters-only model (**B**), the relationship between pseudowords valence and the mean valence of the closest orthographic neighbor(s) (**C**). (Color figure online)

The full model, which performed best in Experiment 1, had an *R*^2^ = .10 and an AIC = −3,138, indicating that the observed and the predicted valence indexes were moderately correlated, *r* = .31 (Fig. 3A). Results of all the linear models tested are reported in Table 2 and showed that the letters-only model outperformed all other models (Fig. 3B). For examples of the most positive and negative stimuli according to the different models we tested, see Table 3.

**Table 2:** Results of the models tested in addition to the full model (using an α = .01, the critical two-tails *r* value for 1,500 observations is = .06) in Experiment 2A

*Model*	*r*	*R*^2^	*AIC*
Letters	.42*	**.18**	**−3277**
Bigrams	.26*	.07	−3087
Dimensions	.34*	.12	−3170
Letters + Bigrams	.19*	.04	−3035
Letters + Dimensions	.35*	.13	−3185
Bigrams + Dimensions	.34*	.12	−3164
Full model	.31*	.10	−3138

The letters-only model outperformed all the other models (including the best model found in Experiment 1)

**Table 3:** Examples of some of the most negative and most positive pseudowords as collected in Experiment 2A, Experiment 3, and as predicted by the letters model, the full model, and the models including the valence of the closest orthographic or semantic neighbor(s) (on the sample of 1,500 items used in Experiment 2A)

Experiment (human ratings)				Model predictions							
2A		3		Letters		Full		Orthographic		Semantic	
Negative	Positive	Negative	Positive	Negative	Positive	Negative	Positive	Negative	Positive	Negative	Positive
‍toutured	boppies	disdorn	mavel	disrusts	zauze	xexen	dapgel	toutured	grendness	unstyps	parseak
‍impails	lalal	‍toutured	flowesh	dirursts	wazo	xeras	avol	plynching	smutscap	dispames	eemild
‍divords	fiffy	‍impails	emperk	disgraud	vevem	xequa	cleb	smapsick	pralse	rauds	corrifit
‍disputs	appite	disspost	upgeer	misdalds	zerow	dyish	ralp	crile	blelshed	caldness	preepy
‍disdorn	mavel	inchained	lalal	sugjucts	whez	xetob	jirm	debty	succempts	nyps	disgraked

## Experiment 2B

In Experiment 2A, the letters-only model outperformed the other models. This suggests that participants were judging the valence of pseudowords based only on surface-level form characteristics, possibly sound-related (see Adelman et al., 2018). However, there remains the possibility that participants might not have been assessing pseudowords’ valence directly, but rather associated orthographically/semantically similar existing words and relied on their valence. To test for this possible effect, we thus re-analyzed the present data in Experiment 2B.

### Methods

#### Orthographic and semantic neighbors’ computation

The method we are applying here requires using actual (i.e., observed) word valences. We thus used the Warriner and colleagues (Warriner et al., 2013) database as a reference with the same set of 13,787 words used in Experiment 1.

In order to retrieve both orthographic and semantic neighbors of our pseudowords, we followed the same method. Specifically, we first created a 1,500 × 13,787 matrix filled with the orthographic or semantic distances between each of the 1,500 pseudowords and each of the 13,787 words. Then, for each pseudoword we selected the word(s) with the lowest distance and retrieved its (their) valence in the Warriner and colleagues (Warriner et al., 2013) database.

Orthographic distance was indexed as Levenshtein distance, which measures the orthographic distance between two strings of symbols by quantifying the minimum number of single-character edits (e.g., insertions, deletions, or substitutions) required to change one element into the other. The Orthographic distance was computed using the *stringdist* R package (Van der Loo, 2014). The method used was the standard *stringdist* method, that is the optimal string alignment (restricted Damerau–Levenshtein distance).

Semantic distance was computed as cosine distance = 1 − cosine similarity between vectors (i.e., transforming it to a distance scale: the lower the value, the closer the two vectors). Word and pseudoword vectors were retrieved from *fastText* (Bojanowski et al., 2017; Mikolov et al., 2017), and distances were computed using the *dist* function of the *proxy* R package (David & Buchta, 2021). After an inspection of the closest semantic neighbors, we noticed that the large majority of the pseudowords had “skijump” or “nylong” as closest neighbors. This was due to the fact that these words are not very embedded in the semantic space (i.e., very similar in their form to out-of-vocabulary strings of letters).^3^ Semantic distance was then computed excluding these two stimuli.

When a pseudoword had more than one neighbor (which only occurs in the orthographic distance set), a mean valence index was computed across the neighbors. This valence was then used to predict the observed valence of the pseudoword. Due to the nature of these two indexes (i.e., the cosine is fully continuous within its range, while Levenshtein distance is limited to integer numbers), strings of letters tend to have only one closest semantic neighbor, but in most cases multiple orthographic ones.

### Results

**Fig. 4:**
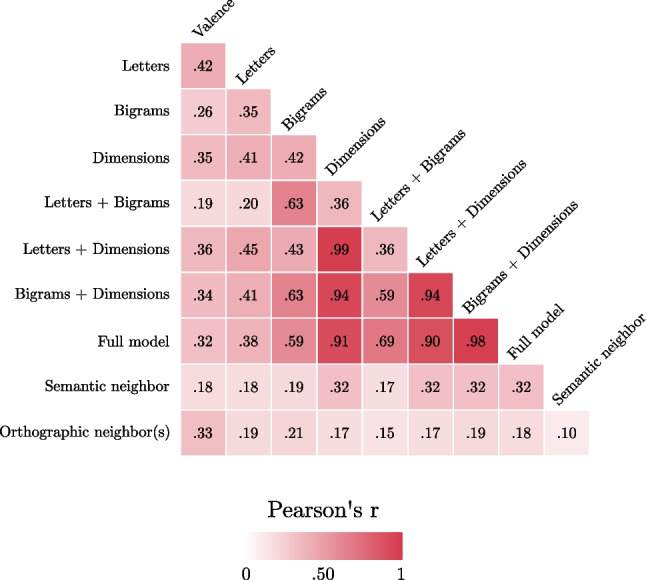
Correlation plot of the pseudowords’ predicted valence scores using different sets of predictors included in Experiment 2A and Experiment 2B as well as of the observed valence (left column). Notably, semantic neighbor valence and orthographic neighbor(s) valence show low correlation index with the other predictors included

A correlation plot of all the predictors included in Experiment 2A and Experiment 2B, as well of the observed valence, is reported in Fig. 4. The observed valence of the pseudowords was predicted across two different linear models, including the valence of the closest orthographic or semantic neighbor(s) as the only (continuous) predictor respectively. The orthographic neighbor(s) model had an *R*^2^ = .11 and an AIC = −3,155, *r* = .33 (Fig. 3C); while the semantic neighbor model had an *R*^2^ = .03 and an AIC = −3,027, *r* = .17, all *p*s < .01. Thus, both indexes can predict pseudowords’ valence to some degree, but the letters-only model from Experiment 2A still clearly performs best (*R*^2^ = .18, AIC = −3,277, *r* = .42).

Next, we evaluated whether adding orthographic and/or semantic neighbor(s) valences to the letters only model provides better estimates. To this end, we estimated three linear models having the three sets of predicted valences combined.^4^ Results are reported in Table 4.

**Table 4:** Results of the models tested in addition to the letters only model (using an α = .01, the critical two-tails *r* value for 1,500 observations is = .06) in Experiment 2B

*Model*	*r*	*R*^*2*^	*AIC*
Baseline (letters model)	.42*	.18	−3277
Orthographic neighbor(s)	.33*	.11	−3155
Semantic neighbor	.17*	.03	−3027
Letters + Orthographic neighbor(s)	.49*	.24	−3399
Letters + Semantic neighbor	.43*	.18	−3294
Letters + Orthographic + Semantic neighbor(s)	.50*	**.25**	**−3411**

The model including all the three sources of valence model outperformed all the other models (including the best model found in Experiment 2A)

The model including all the three sources of valence significantly outperforms the others, *F*(2, 1498) = 72.3, *p* < .001, for the comparison with the baseline model, *F*(1, 1496) = 14.2, *p* < .001, for the comparison with the Letters + Orthographic neighbors model. In this model, all the three predictors were found to be significant, *t* = 15.55, *p* < .001, β = .03, for the letters index, *t* = 11.12, *p* < .001, β = .02, for the orthographic index and *t* = 3.77, *p* < .001, β = .007, for the semantic index.

## Experiment 3

Experiment 2B shows that, even though considering orthographic and semantic neighbors in addition to letters information explains participants’ judgments better, basic surface-level letters information remains the single most prominent predictor. This might however be due to the task at hand: Participants were just instructed to judge whether the *(pseudo)words* cause positive or negative emotions. This does not specify whether participants should consider possible meanings or could simply focus on the form level (“how good/pleasant do the words sound”). Thus, we may observe very different patterns if participants are actively induced to focus on meaning and provide a definition of the word’s meaning alongside their valence judgments. In Experiment 3, we test for this possibility.

### Methods

#### Participants

For this experiment, 111 U.S. participants were enrolled in the experiment through the Prolific online testing platform and tested online. Ten participants were removed because they did not reach the accuracy threshold in our catch trials (see Experiment 2A) or because the written definitions (see below) of the pseudowords that they provided were not adequate (e.g., writing “no meaning,” “gibberish,” “no clue”; see below). The final sample included 101 participants (63 males, 34 females, four nonbinary, *M* age = 36.75 years, *SD* = 10.48, age range: 18–66). The sample size was determined a priori in the same way as described for Experiment 2A, only with a different number of items and trials per participant (see below).

All participants were native English speakers and were naïve to the purpose of the study. Informed consent was obtained from all participants before the experiment. The protocol was approved by the psychological ethical committee of the Humboldt University of Berlin (2020-47) and participants were treated in accordance with the Declaration of Helsinki. Participants were paid £5.66 for their participation in the experiment.

#### Stimuli

Pseudowords included in Experiment 3 were re-sampled from the 1,500 included in Experiment 2A. Since the task requirements and thus the time required to complete the experiment were considerably higher, we opted to reduce the number of pseudowords to 500. The sampling of these 500 pseudowords followed the same rules as described for Experiment 2A, and the final distribution of the predicted valence for the selected items is reported in Fig. 1.

#### Procedure

The procedure was overall similar to Experiment 2A, except for the task instructions and an additional task. In Experiment 3, participants were instructed that they would be shown lists of six (new) words that were names of things, actions or concepts. Their task was to indicate which one of them was the most positive and which one the most negative, focusing on the possible things, actions or concepts these names could refer to. In order to induce participants to focus on these possible meanings, after selecting the most positive and negative items, they were asked to provide a written definition for these two items. These definitions were requested on a separate screen.

In order to keep participants at a good compliance level, we opted to keep the task relatively short, presenting 25 trials to each participant. In total, each participant was presented with a unique set of 31 trials (except for two practice trials that included only words and were shown at the beginning, as well as the additional four catch trials, which all were the same across all participants). All 29 experimental trials were presented in random order after the practice trials. Overall, the task took around 35 minutes to complete.

#### Data analysis

Data analysis was identical to Experiment 2A and Experiment 2B. After transforming the discrete best–worst judgments into continuous valence indexes, we firstly tested for split-half reliability.

Secondly, we tested to which extent the valence indexes of the 500 pseudowords collected in Experiment 3 were correlated with those for the same 500 pseudowords collected in Experiment 2A.

Finally, we predicted these valence indexes across nine linear models, using the seven predicted valences included in Experiment 2A and the additional two included in Experiment 2B.

### Results

The split-half reliability analysis again showed a moderate correlation (of comparable size to Experiment 2A) between the two valence indexes, *r* = .55, *p* < .001. The valence indexes generated in this data collection were highly correlated with those generated in Experiment 2A, *r* = .77.

Results of the linear models tested are reported in Table 5. In contrast to Experiment 2, the best model predicting participants’ performance in Experiment 3 is the one taking the valence of the closest orthographic neighbor(s) to predict pseudoword valence.^5^

**Table 5:** Results of the models tested in addition to the full model (using an α = .01, the critical two-tails *r* value for 500 observations is = .11)

*Model*	*r*	*R*^2^	*AIC*
Letters	.35*	.12	−1078
Bigrams	.27*	.07	−1051
Dimensions	.30*	.19	−1059
Letters + Bigrams	.16*	.02	−1024
Letters + Dimensions	.31*	.09	−1063
Bigrams + Dimensions	.30*	.09	−1059
Full model	.27*	.07	−1051
Semantic neighbor	.15*	.02	−1024
Orthographic neighbor(s)	.40*	**.16**	−**1100**

The Orthographic neighbor(s) model outperformed all the other models (included the best model found in Experiment 1)

Following Experiment 2B, we evaluated whether adding letters information (with the letters-only model still being the best of the seven models from Experiment 2A, see Table 5) and/or semantic neighbor valence to the orthographic neighbor(s) valence model provides better estimates. Results are reported in Table 6.

**Table 6:** Results of the models tested in addition to the one including the valence of the closest orthographic neighbor(s) (using an α = .01, the critical two-tails *r* value for 1,500 observations is = .06) in Experiment 3

*Model*	*r*	*R*^2^	*AIC*
Baseline (*Orthographic neighbor(s) model*)	.40*	.16	**−**1100
Letters + Orthographic neighbor(s)	.49*	**.24**	**−1149.8**
Orthographic + Semantic neighbor(s)	.42*	.18	**−**1109
Letters + Orthographic + Semantic neighbor(s)	.50*	.25	**−**1151.4

The model including letters information and valence of the closest orthographic neighbor(s) can be considered as the best one in explaining the process at hand

The model comparison revealed that the best model is the one comprising letters information and valence of the closest orthographic neighbor(s), *F*(1, 497) = 78.5, *p* < .001 (as compared with the baseline model). In this model, both predictors were significant, *t* = 7.34, *p* < .001, β = .025, for the letters index, and *t* = 8.85, *p* < .001, β = .03, for the orthographic index. Additionally including the valence of semantic neighbor(s) as a predictor does not lead to significantly better model predictions, *F*(1, 496) = 3.57, *p* = .059.

### Discussion

**Fig. 5:**
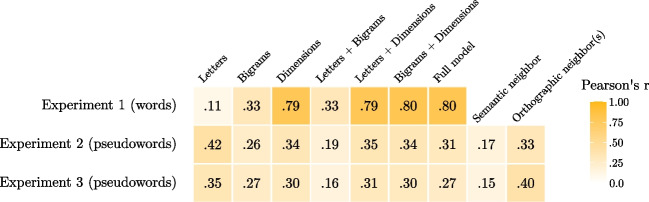
Heatmap showing the *r* coefficients of the models estimated across the three experiments on words and pseudowords valence indexes

In the present study we investigated whether participants can reliably assign valence to pseudowords (using the best–worst rating technique; Hollis, 2018) and which surface-level (i.e., form-based), orthographic, and semantic factors explain participants’ behavior. Across three experiments, we firstly trained a series of models able to predict valences of existing words, indexing different components (i.e., letters, bigrams, semantic dimensions, and their combinations). We used these models to estimate predicted valence scores for pseudowords and identified the best model in explaining the observed ratings for pseudowords (see Fig. 5 for a heatmap of the observed effects). By using this setup, that is by indexing the processes that are at play in words valence and then predicting pseudowords valence, we investigated to what extent humans’ ability to assign valence to novel stimuli can be traced back to surface-level or semantic information as emerging from already mapped information.

Experiment 1 showed that a large portion of the variance (i.e., 65%) in valence judgments for existing words can be explained using letters and bigrams information in addition to semantic dimensions, in line with previous evidence (Adelman et al., 2018; Hollis et al., 2017). Results in Experiment 2A showed that participants were able to reliably indicate pseudowords valence (with moderate convergence between different speakers), and the best model to predict this data relied on letters information only. In Experiment 2B, we tested the valence of the closest orthographic and/or semantic neighbor(s) as additional potential sources of pseudoword valence. Results showed that the model containing all three predictors (letters, orthographic neighbors, and semantic neighbors) was the one explaining more variability in the observed data. Finally, in Experiment 3, we asked participants to describe the possible meanings of the stimuli chosen as best and worst in each trial to induce a focus on word meaning rather than form. We again observed reliable valence ratings for pseudowords, and found that the best performing model predicted these scores relying on letters information and the valence of the closest orthographic neighbor(s).

Taken together, these results indicate that humans can reliably assign valence to pseudowords, word stimuli that are ostensibly meaningless. In doing so, humans would predominantly rely on surface-level information, like the letters included in the words, and secondarily on (orthographically and semantically^6^) similar existent words already mapped in their vocabulary. The dominant letter effect indicates form of the word as a stimulus itself, rather than deeper meaning components, turn out to be the most important factor deciding its perceived valence. Note that we decided to include letters and bigrams (i.e., objective components) instead of phoneme information as the data were collected in English, a nontransparent language. Thus, in contrast to existing words, the exact pronunciation of each pseudoword could not have been established with certainty. Other studies employing phonemes information provided results consistent with the possibility that the phonetic component could play a role (Adelman et al., 2018; Aryani et al., 2018). Further investigations are therefore required to test such effects of other linguistic components (primarily related to sound and phonology) that could not be handled in the present study. A promising avenue would be to repeat our study in a language with transparent grapheme-to-phoneme translation such as Spanish or Italian. This would also have the additional benefit of testing the generalizability of our results across different languages, thus addressing another limitation of the present study.

Notably, the model tracing valence back (also) to semantic dimensions as extracted from *fastText* was not found to be the best performing model even in Experiment 3, where participants were explicitly instructed to rely on (possible) meanings of the pseudowords while performing the task. While we cannot definitively rule out that this source of information might play some role in determining pseudoword valence, our results indicate that speakers mainly rely on information carried by the letters that compose the stimulus and on the (orthographic) similarity with known words. However, our results still provide evidence for humans’ natural inclination in trying to make sense of (linguistic) experience even when it carries no clear meaning, as evidenced by the fact that speakers explore these orthographic neighbors (whose valence is a function of the valence of their *referents*) and even their semantic neighbors (Experiment 2B) to inform their judgments. As additional remark on this, it should be noted that different models (e.g., employing BERT-like or GPT-like Large Language Models), model parameters (e.g., manipulating *n*-grams length), corpus characteristics (e.g., training the model on Wikipedia, on the Corpus of Contemporary American English) or type of item could lead to different results with respect of the (possible) semantic processes involved: As an example, in a very recent study, Joosse et al., 2024, successfully predicted the polarity (good vs. evil) of fictional characters based on their names using a *fastText* model.

The methodology employed here could be straightforwardly applied to semantic dimensions other than valence, which would allow to test for the specific contributions of surface-level, orthographic and semantic processes to different meaning components. One obvious candidate would be the other main semantic and emotional dimension, arousal (Osgood et al., 1957). As compared with valence—which can be conceived as involving higher-order cognitive and evaluative processes—arousal involves more automatic and perceptual reactions. As a consequence, arousal ratings might be even more susceptible to immediately available surface-form information than valence ratings (for evidence on words processing, see Aryani et al., 2016, 2018).

On a more fundamental level, by predicting pseudowords valence using indexes extracted from existing word data (Experiment 1), we directly show that participants were relying on already mapped information when trying to make sense of these novel (and apparently meaningless) stimuli. That is, humans would possibly judge the valence of these stimuli based on statistical regularities they have been exposed to (i.e., based on previous linguistic experience). This does not mean that humans rely on the exact same information when assigning valence to words and pseudowords, and different processes can be at play when making these judgments: for example, words valence can be retrieved from semantic memory, while estimates pseudowords valence have to be newly constructed. However, our results still indicate that humans do assign valence to pseudowords by relying on already mapped information—namely, letters information as extracted from the form-meaning regularities (more specifically, form-valence regularities) in *existing words* only (as estimated in Experiment 1). Notably, our results can be seen as complementary with previous psychological studies employing fastText vector-based representations (e.g., Gatti et al., 2023; Pugacheva & Günther, 2024). For example, Gatti and colleagues (Gatti et al., 2023) found that the same mechanisms governing the semantic processing of words can also subserve pseudowords processing. In interpreting our findings, we want to highlight that also in the present study we find commonalities between the processing of words and pseudowords, but these remain at the word-form level and do not extend to the activation of semantic representations. However, our present results do not allow the conclusion that no semantic representations are activated in pseudoword processing: Valence is only one component of meaning, which at least for words only manifests in some of the distributional vector dimensions (Hollis & Westbury, 2016). Even when semantic representations for pseudowords are routinely activated during processing, this specific information might be absent from these representations or too weak to predict specific semantic phenomena such as valence (at least in comparison to other sources of information such as form features); but nonetheless, the overall semantic similarity (taking into account all semantic dimensions) between activated semantic representations may at the same time still predict phenomena such as priming (Gatti et al., 2023).

These findings can be further framed within non-arbitrary perspectives on language processing (i.e., systematic form-meaning mapping; Dingemanse et al., 2015). The surface-level letters index, as well as participants’ reliance on the valence of existing words can be explained referring to humans’ tendency to detect systematic and statistical regularities in the (language) environment (Romberg & Saffran, 2010; Vidal et al., 2021). Consistent with this, previous studies have shown that humans are able to exploit these mechanisms across a broad range of linguistic processes, in the grammatical, orthographical and phonological, and even semantic domain (for a review, see Bogaerts et al., 2021; Christiansen, 2019). Integrating these results, our findings indicate that these experience-learning mechanisms can also be exploited when trying to assign valence to novel (word) stimuli.

In conclusion, by training a model on existing words and using it to predict valence judgments for pseudowords, we provide a data-driven account of the processes at play when assigning valence to novel stimuli. Our findings support perspectives on the non-arbitrariness of language and provide insights regarding how humans process the valence of novel words. On the practical side, our observations that some labels are inherently and systematically more appealing than others have direct implications whenever there is the need to engineer a (new) label for something, which can have commercial applications when designing appealing brand or product names, or social relevance when creating labels for groups or individuals.

## Web interface

At this link: http://danielegatti.shinyapps.io/pseudoval we provide a free web interface named QUOKY that allows to estimate the valence of a given pseudoword according to the three best models resulting from the present study. Specifically, typing a pseudoword, it is possible to obtain an estimated valence index according to (i) the letters only model as emerging from Experiment 2, (ii) the valence of the closest orthographic neighbor(s) as emerging from Experiment 3, and (iii) the additive effect of these two components as emerging from Experiment 3.

## Supplementary Information

Below is the link to the electronic supplementary material.

Supplementary file1 (DOCX 132 KB)

## Data Availability

All data, scripts and codes used in the analysis are available at: https://osf.io/kv9at/. Here: http://danielegatti.shinyapps.io/pseudoval, we also distribute an R Shiny app named QUOKY that can be freely used to estimate the valence of pseudowords based on the best performing models as resulting from the experiments performed here. This study was not preregistered.

## Footnotes

1 Note that *cupgel* (as well as other similar stimuli) was later on removed as it is a pseudocompound (i.e., a pseudoword composed of two existing words).

2 As Supplementary Material we also describe the results obtained using a full model predictor computed using an ElasticNet model as well as using a *fastText* model including 3- to 6-grams. None of these predictors performs better than the best one(s) included here.

3 Applying an isoMDS (i.e., a procedure that estimates *x-* and *y*-coordinates starting from a matrix of distances, see: Venables & Ripley, 2002) we indeed found that words and pseudowords occupy different sections of the space, with these two words being completely embedded in the pseudowords section. This result can be interesting in interpreting the results of other studies (although note that the isoMDS forces 300-dimensional representations into 2-dimensionsal ones) and we believe it is mainly driven by the fact that word representations include the full vector in addition to the *n*-gram vectors.

4 Note that in this case the models tested were for example:

$$\begin{array}{l}Valence \sim Letter{s}_{valence}+Orthographic\_neighbor{s}_{valence}\\ Valence \sim Letter{s}_{valence}+Semantic\_neighbo{r}_{valence}\end{array}$$

5 As an additional sanity check we tested whether this effect could have been item-dependent. That is, we reanalyzed the observed valence collected in Experiment 2A for this subset of 500 pseudowords. Results showed that when using the valence data collected in Experiment 2A, the letters model was still the best one.

6 It should be noted that the effect found for the valence of the closest semantic neighbor supports previous evidence regarding pseudowords’ semantics (i.e., it would not be possible to find an effect related to semantics without being able to retrieve semantic information).

## Declarations

### Prior dissemination

A previous version of this article was uploaded on PsyArXiv (https://psyarxiv.com/sfzgr)
