# Supplementary material for: Valence without meaning: Investigating form and semantic components in pseudowords valence
Source: Psychon Bull Rev. 2024 Apr 2;31(5):2357–69. doi: 10.3758/s13423-024-02487-3 (PMC11543720; doi:10.3758/s13423-024-02487-3)
Supplement: Supplementary file 1 — Supplementary file1 (DOCX 132 KB) [file 13423_2024_2487_MOESM1_ESM.docx]

**Supplementary Experiment 1**

**Methods**

In Supplementary Experiment 1 we reanalyzed the data collected in Experiment 2A and Experiment 3 re-estimating a letters only model on the pseudowords data collected and combining this new index with orthographic and/or semantic neighbor(s) valences.

**Data analysis and results**

Following Experiment 1 procedure, we firstly estimated the letters only model starting from pseudowords information. That is, we estimated a linear model including only single-letter information, with 26 predictors.

Then, after having estimated that model, using the *predict* R function we estimated a valence index and predicted pseudowords valence using this index. Results showed that estimating these new models on both Experiment 2A and Experiment 3 provided better predictions of pseudowords valence. Specifically, on the entire set of 1,500 pseudowords tested in Experiment 2A, the model had *r* = .55 (*R^2^* = .30), AIC = -3524; while, on the set of 500 pseudowords tested in Experiment 3, the model had *r* = .49 (*R^2^* = .24), AIC = -1149.

Then, we evaluated whether adding orthographic and/or semantic neighbor(s) valences to these models provides better estimates. That is, in order to test for the added complexity, we estimated for each experiment three linear models having the three sets of predictors combined.

Results are reported in Table S1. Across both re-analyses the models including letters, valence of the closest orthographic neighbor(s) and of the closest semantic neighbor outperforms the others (Figure S1A and S1B). These results indicate that across both tasks participants are likely relying on multiple sources of information, ranging from pure form-level information to orthographic and semantic information as extracted from existing words.

Table S1. Results of the models tested Experiment 4 on the data collected in Experiment 2A and Experiment 3. Across both re-analyses the model including letters, valence of the closest orthographic neighbor(s) and of the closest semantic neighbor outperforms the others.

|  | ***Model*** | ***r*** | ***R^2^*** | ***AIC*** |
| --- | --- | --- | --- | --- |
| Experiment 2A | Letters | .55* | .30 | -3524 |
|  | Letters + Orthographic neighbor(s) | .60* | .36 | -3671 |
|  | Letters + Semantic neighbor | .56* | .31 | -3546 |
|  | Letters + Orthographic + Semantic neighbor(s) | **.61*** | **.37** | **-3688** |
| Experiment 3 | Letters | .49* | .24 | -1149 |
|  | Letters + Orthographic neighbor(s) | .60* | .36 | -1237 |
|  | Letters + Semantic neighbor | .49* | .24 | -1152 |
|  | Letters + Orthographic + Semantic neighbor(s) | **.61*** | **.37** | **-1240** |


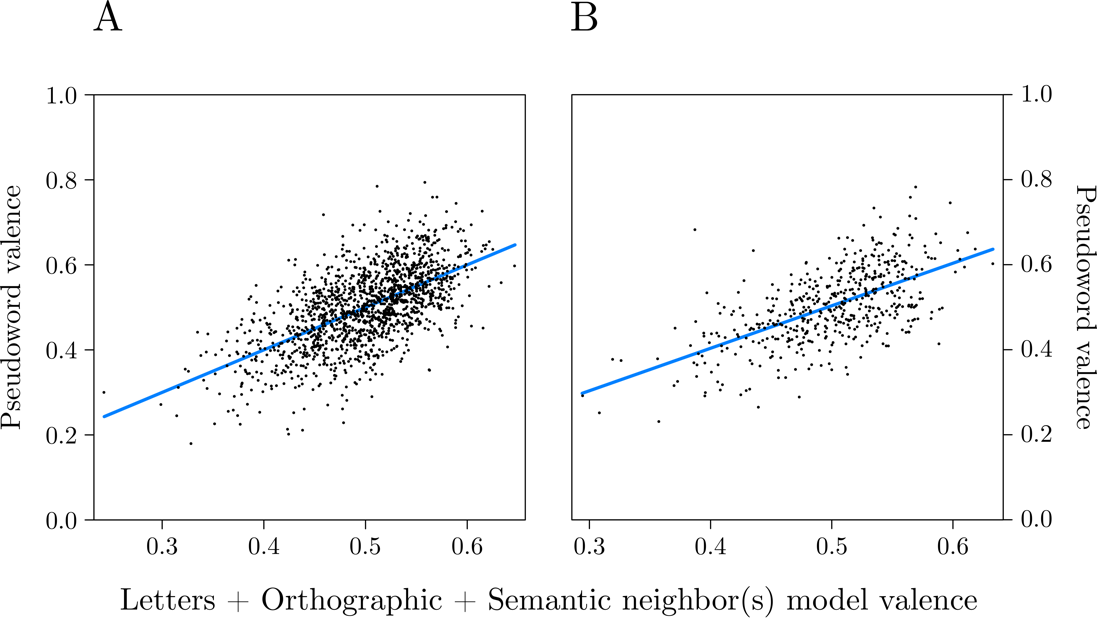


Figure S1. Plots showing the relationship between pseudowords valence and the best performing models (i.e., the ones comprising letters information, valence of the closest orthographic and semantic neighbor(s)) trained on pseudowords information as found in Supplementary Experiment 1 by re-analyzing the data of Experiment 2A (A) and Experiment 3 (B).

**Supplementary Information**

Table S2 includes the estimates for each letter as emerging from the letters model estimated in Experiment 1.

Table S2. Estimates of the model estimated on letters information carried by words in Experiment 1

| ***letter*** | ***b*** | ***p*** |
| --- | --- | --- |
| a | .03 | .06 |
| b | -.07 | .02 |
| c | -.03 | .21 |
| d | -.20 | < .001 |
| e | .07 | < .001 |
| f | -.01 | .66 |
| g | -.05 | .09 |
| h | -.01 | .59 |
| i | -.02 | .36 |
| j | -.07 | .43 |
| k | -.07 | .06 |
| l | < .01 | .85 |
| m | < .01 | .97 |
| n | -.02 | .40 |
| o | .05 | .01 |
| p | -.01 | .82 |
| q | .08 | .39 |
| r | -.06 | < .001 |
| s | -.12 | < .001 |
| t | -.04 | .05 |
| u | -.09 | < .001 |
| v | .02 | .60 |
| w | .01 | .81 |
| x | -.08 | .29 |
| y | .01 | .83 |
| z | .12 | .10 |

Table S3 includes the estimates obtained using a full model predictor computed using an ElasticNet model as well as using a *fastText* model including 3- to 6-grams..

Table S3. Results of additional predictors tested estimated using the ElasticNet method and/or using a *fastText* model including 3- to 6-grams as compared with the best model found across both Experiments (Letters) and the one predicted from Words valence (Full model (4-grams)).

|  | **Predictor** | **Method** | ***r*-coefficient** |
| --- | --- | --- | --- |
| **Experiment 2** | Letters | Linear | **.42** |
|  | Full model (4-grams) | Linear | .31 |
|  | Full model (3- to 6-grams) | Linear | .29 |
|  | Full model (4-grams) | ElasticNet | .33 |
|  | Full model (3- to 6-grams) | ElasticNet | .32 |
| **Experiment 3** | Letters | Linear | **.35** |
|  | Full model (4-grams) | Linear | .27 |
|  | Full model (3- to 6-grams) | Linear | .30 |
|  | Full model (4-grams) | ElasticNet | .29 |
|  | Full model (3- to 6-grams) | ElasticNet | .28 |
